# Diagnostic and prognostic value of blood samples for KRAS mutation identification in lung cancer: a meta-analysis

**DOI:** 10.18632/oncotarget.15972

**Published:** 2017-03-07

**Authors:** Hongchang Shen, Keying Che, Lei Cong, Wei Dong, Tiehong Zhang, Qi Liu, Jiajun Du

**Affiliations:** ^1^ Department of Oncology, Shandong Provincial Hospital Affiliated to Shandong University, Jinan, P.R. China; ^2^ Institute of Oncology, Shandong Provincial Hospital Affiliated to Shandong University, Jinan, P.R. China; ^3^ Department of Thoracic Surgery, Shandong Provincial Hospital Affiliated to Shandong University, Jinan, P.R. China

**Keywords:** lung cancer, blood, KRAS, diagnostic test, predictive factor

## Abstract

Circulating tumor DNA (ctDNA) and tumor cells (CTC) are novel approaches for identifying genomic alterations. Thus, we designed a meta-analysis to evaluate the diagnostic value and prognostic significance of a KRAS proto-oncogene, GTPase (KRAS) mutation for lung cancer patients. All included articles were from PubMed, EMBASE, Web of Science and Cochrane Library. Twelve articles that described 1,131 patients were reviewed. True positives (TP), false positives (FP), true negatives (TN), and false negatives (FN) were used to calculate pooled sensitivity, specificity, the positive likelihood ratio (PLR), the negative likelihood ratio (NLR), a diagnostic odds ratio (DOR), the area under the curve (AUC) and corresponding 95% confidence intervals (95% CI). PLR is calculated as sensitivity/(1-specificity) and NLR is (1– sensitivity)/specificity. DOR is a measured of diagnostic effectiveness (PLR/NLR). A survival analysis subgroup was also designed to evaluate prognostic significance. Pooled sensitivity, specificity, PLR, NLR, DOR and AUC were 0.79 (95% CI, 0.63-0.89), 0.93 (95% CI, 0.89-0.96), 12.13 (92% CI, 7.11-20.67), 0.22 (95% CI, 0.12-0.41), 54.82 (95% CI, 23.11-130.09), and 0.95 (95% CI, 0.93–0.96), respectively. KRAS mutation and wild-type hazard ratios for overall survival and progression-free survival were 1.37 (95% CI, 1.08–1.66), 1.46 (95% CI, 1.15-1.77) in blood samples, and 1.16 (95% CI, 1.03–1.28), 1.28 (95% CI, 1.09–1.46) in tumor tissue.

## INTRODUCTION

Cancer is a serious global public health problem and lung cancer, in particular, is a leading cause of cancer-related death in the United States. In 2016, almost 250,000 new cancer cases will be reported and slightly more than 150,000 deaths will result. [1] Additionally, lung cancer is the chief cause of cancer death among men and the second most common cause of cancer death among women worldwide. [2] Such high mortality is due to lack of early detection using lung cancer markers.

*KRAS*, one of the most frequently mutated oncogenes, contributes to the mitogen-activated protein (MAP) kinase pathway, which controls cell growth and differentiation. [3, 4] The *KRAS* pathway is also involved in the regulation of lung cancer, participating in the downstream signaling network of epidermal growth factor receptor (EGFR). The most commonly mutated codons are 12, 13, and 61 and this causes drug resistance to EGFR tyrosine kinase inhibitors (EGFR-TKIs). Several studies suggest that *KRAS* mutations should be known prior to using EGFR-TKI therapy for lung cancer patients. [5–7]

Although tumor tissue is the reference standard for *KRAS* mutation confirmation, obtaining tissue samples is difficult, costly, and invasive. [8] In addition, most advanced lung cancer patients are unable to tolerate surgical procedures. Thus, a more feasible but accurate method for assaying *KRAS* mutations is needed. Blood testing is less invasive, easily-accessible and can be repeated. [7, 9] Thus, ctDNA and CTCs can be used as a high diagnostic value and prognostically significant source for identifying *KRAS* mutations in lung cancer patients.

## RESULTS

### Search results

**Figure 1:**
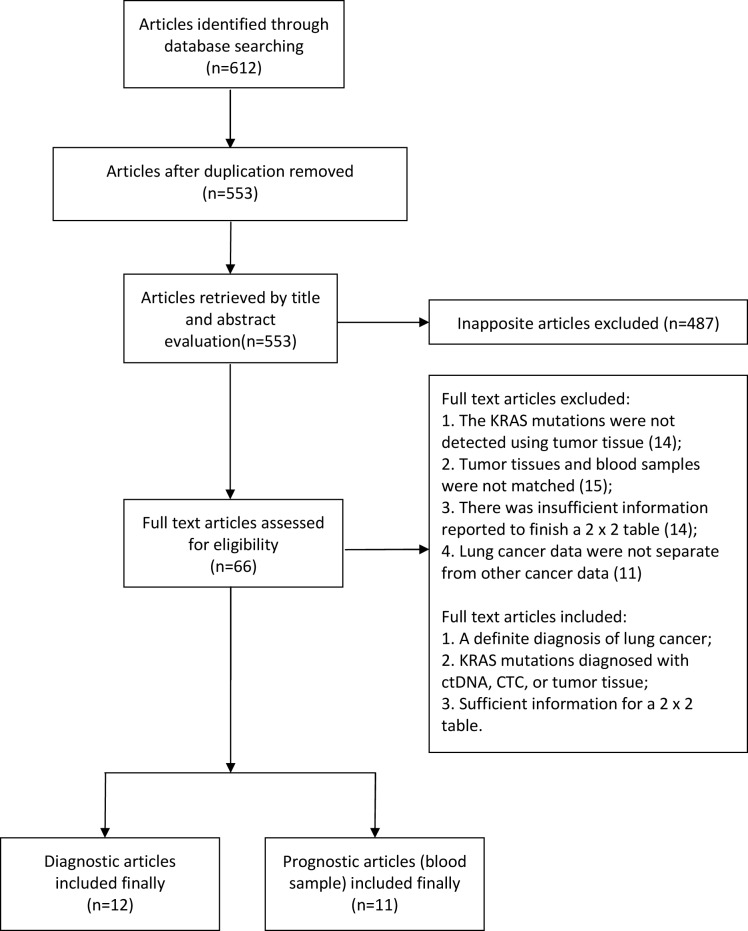
Flow diagram of study inclusion and exclusion for meta-analysis

As shown in Figure 1, our database searched 612 records, of which 59 records were duplicates. After a primary screening of the titles and abstracts, 487 records were excluded. By reviewing full-text articles, we excluded further articles. 12 eligible articles [7, 10–20] with 1131 patients for diagnosis and 11 articles (blood samples) [7, 12, 21–26] for prognosis were included in this meta-analysis. We also included 15 studies in which the *KRAS* mutation was detected by tumor tissue for prognostic subgroup analysis. [27–40]

### Baseline characteristics of identified studies

**Figure 2:**
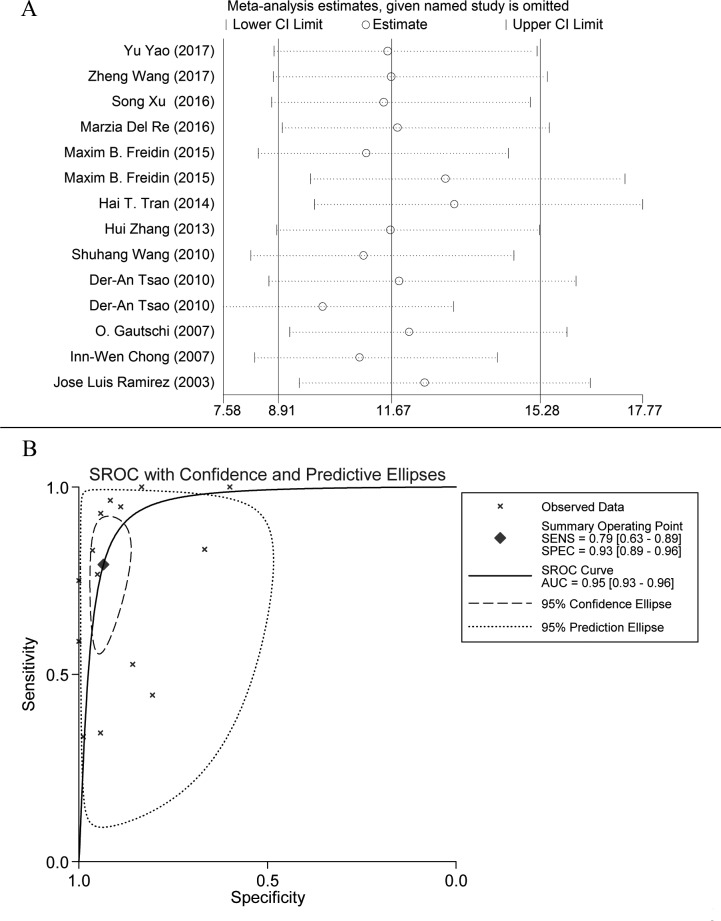
**A**. Sensitivity analysis plot of meta-analysis. Every row represents an included study. The width of the horizontal line represents the 95% CI for each study. The vertical bar on both sides represents the lowest and highest values of 95% CI. **B**. SROC curve: each X mark represents a study and AUC is the area under the curve.

Baseline characteristics of eligible studies are shown in Table 1. The included articles were published between 2003 and Jan, 2017. Two articles had more than one combination of statistics. [13, 15] CTC were detected by two methods in Der-An Tsao's article; and ctDNA and CTC were both detected in the article of Maxim B. Freidin. Most of the included lung cancer patients were at III/IV TNM stage with adenocarcinoma of the lung. All describing 1,131 subjects were included. Characteristics of eligible studies appear in Table 1. A QUADAS-2 plot is shown in Supplementary Figure S1, and sensitivity analysis is presented in (Figure 2A) which was accomplished by excluding studies one by one. Data were stable and were not significantly different.

**Table 1:** Characteristics of eligible studies

Author(year)	Country	Number	Female	Smoker	AC	treatment	sample	Detection methods	TNM(I/II/III/IV)
Yao (2017)	China	39	51.30%	25.60%	87%	Frozen or FFPE	Plasma	NGS	0/0/8/31
Wang (2017)	China	103	53.40%	32%	100%	FFPE	Plasma	cSMART	0/0/25/78
Xu (2016)	China	42	45.20%	NA	69.00%	FFPE	Plasma	NGS	0/0/27/15
Del Re (2016)	Italy	8	60.60%	33.30%	NA	NA	Plasma	ddPCR	0/0/1/32
Freidin (2015)^a^	England	82	45.10%	NA	57.40%	FFPE	Plasma	COLD-PCR	27/9/8/31
Freidin (2015)	England	82	45.10%	NA	57.40%	FFPE	Peripheral blood	HRM	27/9/8/31
Tran (2014)	America	154	NA	NA	NA	NA	Plasma	COLD-PCR	NA
Zhang (2013)	China	86	43.00%	51.20%	75.60%	FFPE	Plasma	MEL	0/0/16/70
Wang (2010)	China	273	42.10%	58.60%	72.50%	FFPE	Plasma	RFLP-PCR	0/0/74/199
Tsao (2010)a	Taiwan, China	209	NA	NA	NA	Frozen	Peripheral blood	CLMA	NA
Tsao (2010)	Taiwan, China	209	NA	NA	NA	Frozen	Peripheral blood	WCHMA	NA
Gautschi (2007)	Switzerland	9	30.60%	69.40%	43.90%	FFPE	Plasma	RFLP-PCR	15/11/63/91
Chong (2007)	Taiwan, China	76	57.90%	46.10%	72.40%	Frozen	Peripheral blood	membrane array analysis	10/21/20/25
Ramirez (2003)	Spain	50	4.00%	98.00%	30.00%	Frozen	Serum	methylation-specific PCR	6/11/18/5

AC: adenocarcinoma; NA: not available; FFPE: formalin-fixed paraffin embedded; NGS: next generation sequencing; cSMART: circulating single-molecule amplification and resequencing technology; ddPCR: droplet digital PCR; COLD-PCR: co-amplification at lower denaturation temperature-PCR ; HRM: high resolution melting; MEL: mutant enriched liquid chip; RFLP-PCR: restriction fragment length polymorphism PCR; CLMA: colorimetric membrane array method ; WCHMA: weighted chemiluminescent membrane array.

^a^: For studies by Freidin and Tsao's group, KRAS mutation was measured in ctDNA and CTC, and ctDNA and CTC data were analyzed as two independent studies.

### Diagnostic accuracy of KRAS mutation in blood samples

**Figure 3:**
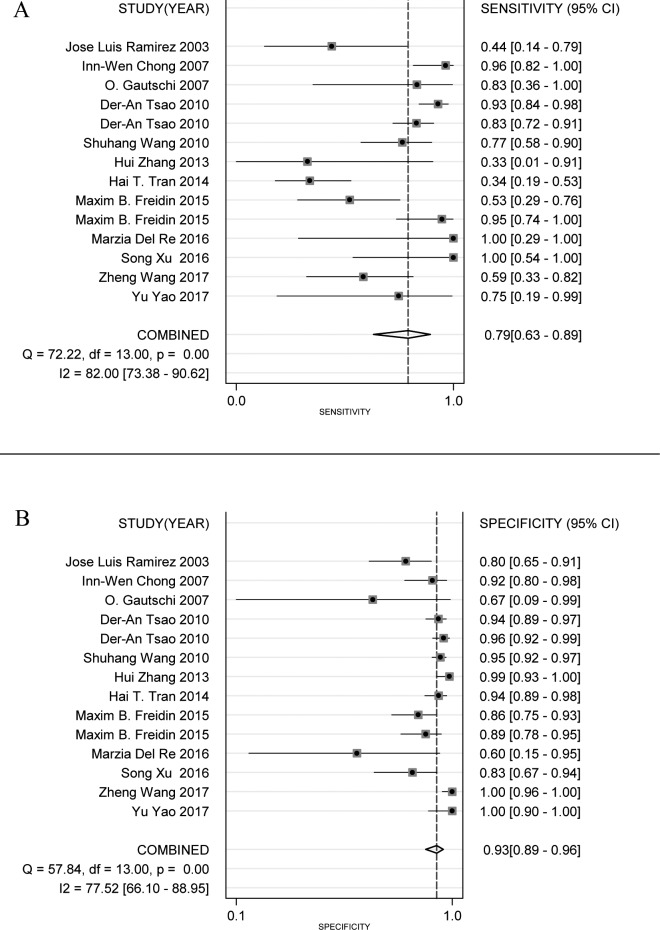
Forest plots of sensitivity (**A**) and specificity (**B**) for blood samples (ctDNA and CTC). The width of the horizontal line represents the 95% CI of each study, square proportional means the weight of every study. The weight is evaluated by the sample size and is presented as percent of total. The diamond represents pooled sensitivity, specificity and 95% CI.

**Figure 4:**
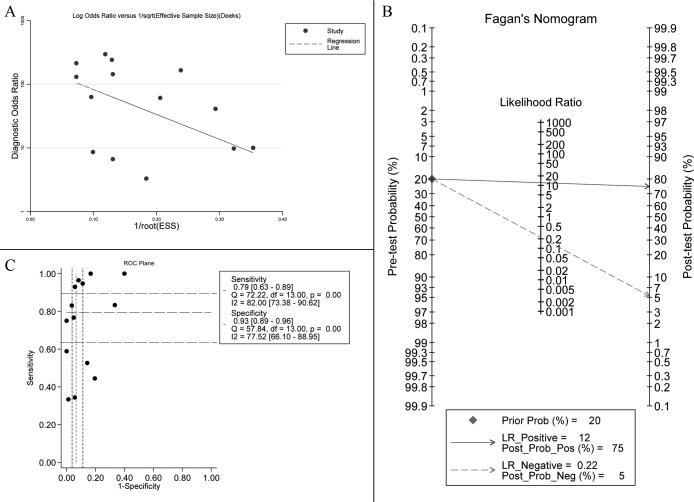
**A**. Deek's funnel plot indicates no significant publication bias (*p* = 0.218 > 0.05). **B**. Fagan's Nomogram of blood samples for *KRAS* mutation identification. **C**. ROC plane for threshold effect. Each black spot represents an included study and does not constitute a “shoulder shape” graph, which represents no significant threshold effect.

**Figure 5:**
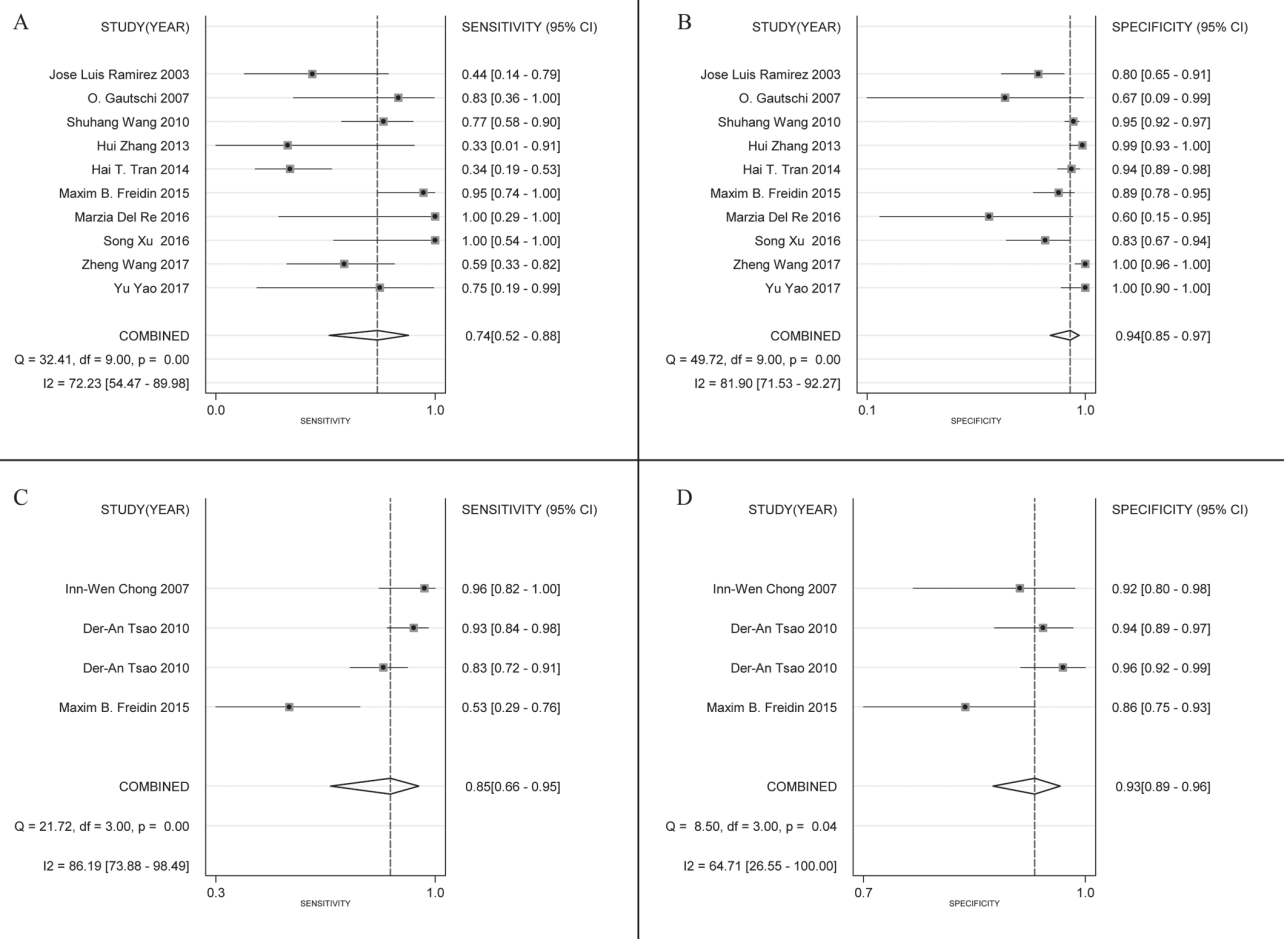
Forest plots of sensitivity and specificity for ctDNA (sensitivity, **A**; specificity, **B**) and CTC (sensitivity, **C**; specificity, D). The width of the horizontal line represents the 95% CI of each study, square proportional means the weight of every study. Weight is evaluated by sample size and presented as percent of total. Diamond represents pooled sensitivity, specificity and 95% CI.

Figure 3 shows a Forest plot of the meta-analysis. The pooled sensitivity of blood samples for the detection of *KRAS* mutation was 0.79 (95% confidence interval (CI), 0.63-0.89) and pooled specificity was 0.93 (95%CI, 0.89-0.96). Table 2 shows that pooled PLR was 12.13 (92%CI,7.11-20.67),, NLR was 0.22 (95%CI, 0.12-0.41) (Figure S2), DOR was 54.82 (95%CI, 23.11-130.09), and AUC was 0.95 (95%CI, 0.93-0.96) (Figure 2B). As the Fagan's nomogram is shown in Figure 4B), PLR was 12, NLR was 0.22 and post-test probability were 75 and 5, respectively, indicating that blood samples are reliable for measuring *KRAS* mutations. Data show that *KRAS* mutations can be assayed with high diagnostic accuracy and specificity. Figure 5 shows a Forest plot of ctDNA and CTC. The pooled sensitivity of ctDNA was 0.74 (95%CI, 0.52-0.88) (Figure 5A), while the pooled specificity was 0.94 (95%CI, 0.85-0.97) (Figure 5B). The pooled sensitivity of CTC was 0.85 (95%CI, 0.66-0.95) (Figure 5C), and the pooled specificity was 0.93 (95%CI, 0.89-0.96) (Figure 5D).

**Table 2:** Subgroup analysis of KRAS mutation in blood sample

Subgroups	Patientsa	Sensitivity	Specificity	PLR	NLR	DOR	AUC
Total	1422	0.79 (0.63-0.89)	0.93 (0.89-0.96)	12.13 (7.11-20.67)	0.22 (0.12-0.41)	54.82 (23.11-130.09)	0.95 (0.93-0.96)
Race	1422						
Asian	1037	0.84 (0.68-0.93)	0.96 (0.92-0.98)	22.02 (11.66-41.57)	0.17 (0.08-0.34)	132.57 (72.86-241.23)	0.97 (0.95-0.98)
Caucasian	385	0.71 (0.42-0.89)	0.87 (0.78-0.92)	5.354 (3.207-8.937)	0.334 (0.142-0.784)	16.027 (4.953-51.858)	0.89 (0.85-0.91)
Detection method	1422						
ctDNA	846	0.74 (0.52-0.88)	0.94 (0.85-0.97)	11.68 (5.19-26.27)	0.28 (0.14-0.55)	41.79 (14.48-120.60)	0.93 (0.90-0.95)
CTC	576	0.85 (0.66-0.95)	0.93 (0.89-0.96)	12.72 (6.75-23.96)	0.16 (0.06-0.41)	81.188 (18.246-361.266)	0.96 (0.94-0.97)
Treatment in tissue	1221						
FFPE	677	0.76 (0.56-0.89)	0.94 (0.85-0.98)	12.68 (5.36-30.02)	0.26 (0.13-0.50)	49.38 (17.81-136.87)	0.93 (0.90-0.95)
Frozen	544	0.85 (0.63-0.95)	0.93 (0.86-0.96)	11.71 (5.38-25.49)	0.16 (0.06-0.47)	71.88 (12.75-405.10)	0.95 (0.93-0.97)

^a^: For studies by Freidin and Tsao's group, KRAS mutation was measured in ctDNA and CTC, and ctDNA and CTC data were analyzed as two independent studies.

### Sub-groups

Sub-group analysis is shown in Table 2. Race, detection method and treatment are displayed and data show that Asian subjects experienced greater diagnostic accuracy compared with Caucasians. CTC and frozen tissue was more sensitive than ctDNA and FFPE.

### Outcomes

**Figure 6:**
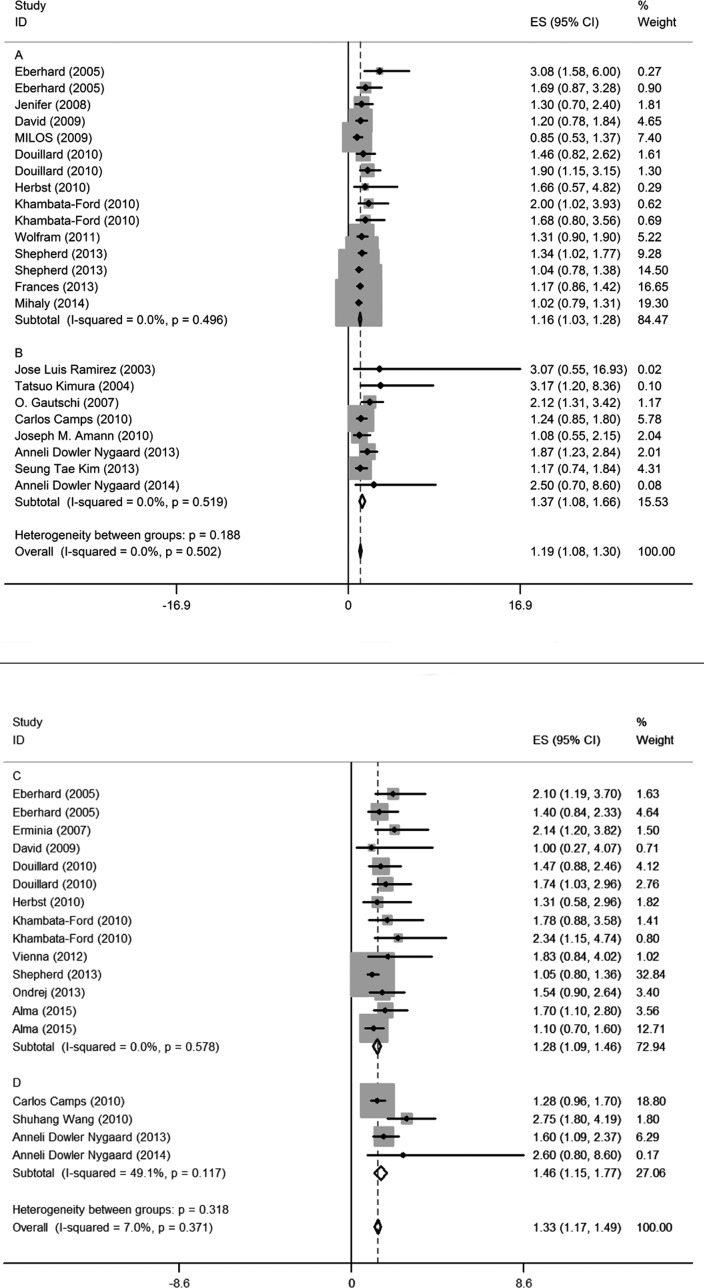
Forest plots of pooled HR for OS (tumor tissue: **A**, blood sample: **B**) and PFS (tumor tissue: **C**, blood sample: **D**) comparing patients of KRAS mutations with wild-type *KRAS*. The width of horizontal line represents the 95% CI of each study and square proportional means the weight of every study. Weight is evaluated by sample size and is presented as percent of total. Diamond represents pooled HR and 95% CI.

The estimated pooled HRs for OS and PFS is displayed in Figure 6 and data show that poorer prognosis is correlated with *KRAS* mutations. Subgroup analysis indicated that lung cancer patients with *KRAS* mutations had a significantly shorter OS and PFS compared to wild-type lung cancer patients. Additionally, there was no significant difference between HRs for blood samples and tumor tissues so both can be used.

### Heterogeneity and publication bias

I^2^ values of pooled sensitivity and specificity were 82.00 (95%CI, 73.38-90.62) and 77.52 (95%CI, 66.10-88.95), respectively. For ctDNA, I^2^ of sensitivity and specificity were 72.23 (95%CI, 54.47-89.98) and 81.90 (95%CI, 71.53-92.27). For CTC, I^2^ of sensitivity and specificity were 86.19 (95%CI, 73.88-98.49) and 64.71 (95%CI, 26.55-100.00), which implies a statistically significant heterogeneity. Most heterogeneity was derived from the threshold effect and differenced among studies. The ROC plane and statistical data show no significant threshold effect (Figure 4C). The Spearman correlation coefficient was 0.367 and the *P* value was 0.197 (*P* > 0.05), indicating no significant threshold effect. Therefore, we suspect that heterogeneity is likely rooted in differences among studies. Potential publication bias was evaluated using a Deek regression test (Figure 4A), and no significant publication bias was discovered (*p* = 0.218 > 0.05).

## DISCUSSION

Detecting *KRAS* mutations in lung cancer is useful for predicting patient outcomes and targeting therapy and tumor tissue is currently used for this assay. Limitations to this approach include patient age and health, so a simple, minimally invasive approach for measuring *KRAS* mutations is required and blood sampling may be that solution. To address this issue, we conducted a meta-analysis to evaluate the diagnostic accuracy and prognostic significance of using blood samples for *KRAS* mutation assay. The results show that blood sampling offered high sensitivity and specificity which suggests that *KRAS* mutations can be assayed this way when tumor tissue is inconvenient or unavailable. Also, blood samples offered high diagnostic accuracy. [41, 42] Finally, likelihood ratios and post-test probability are also important testing standards. [43] The value of likelihood ratios ranges from 0 to infinity. When likelihood is 2-5, post-test probability is slightly increased. When likelihood is > 10, post-test probability increases significantly. In this study, PLR was 12 and NLR was 0.22, which clearly changed the post-test probability.

Subgroup analysis to identify factors that can influence diagnostic accuracy included race, detection method, and tissue treatment. Data show that compared with Caucasians, *KRAS* mutations in blood samples of Asians was more accurate and sensitive when using frozen tumor tissue samples and CTC methods compared to FFPE tissue samples and ctDNA. FFPE can lead to a cross-link between proteins and nucleic acids but this did not occurs with nitrogen-frozen tissues. CTC was more sensitive than ctDNA [44], perhaps due to fewer included studies. Detection methods, collection timing, and TNM stage were not analyzed due to too few studies including this information. Subgroup survival analysis indicated that *KRAS* mutations are associated with significant increases in mortality but there were no differences between blood samples and tumor tissues for OS and PFS, which suggest that blood sampling is suitable for replacing tissue assay.

This is the first meta-analysis to evaluate *KRAS* mutations in blood samples for treating lung cancer. Liquid biopsies allow identification of molecular targets, assessment of prognosis, monitoring therapeutic response and molecular profiles in real time as well as diagnosis of disease recurrence or progression. We found that liquid was highly accurate and high ctDNA and CTC are correlated with poorer prognosis for lung cancer patients. [45] Thus, ctDNA and CTC can be used to confirm *KRAS* mutations in lung cancer instead of tumor tissue and suggest details about prognosis. The diagnostic value and prognostic significance of blood sampling for lung cancer patient monitoring is unclear but our data suggest that it is worth investigating.

The meta-analysis has several limitations such as potential publication bias. We used well-selected articles and Supplementary Data and Deek's funnel plot did not confirm statistical significance (*p* = 0.170 > 0.05). Second, some studies were small and this may have caused bias but a sensitivity analysis suggested that sample size did not influence pooled results significantly. Third, significant heterogeneity existed in our meta-analysis and the ROC plane and Spearman correlation coefficient data indicated that heterogeneity was not due to a threshold effect. Thus, heterogeneity may be primarily due to small sample studies [12, 17] and differences among study detection methods. Studies also differed with respect to race, TNM classification, and percent of lung adenocarcinomas. We tried to establish a subgroup for test methods but because we had few studies and varied methods within them, this was difficult. Future studies should be designed to evaluate differences in detection methods. Finally, in the prognostic analysis sub-group, most studies did not provide a HR so we calculated one (at 95% CI using a survival curve) and it may indicate result bias.

In conclusion, lung cancer is a leading cause of cancer-specific mortality around the world and with the rapid development of liquid biopsy, CTCs and ctDNA provide a novel method for assaying *KRAS* mutations in lung cancer. Our meta-analysis indicates that this approach has advantages over other methods and that it is highly specific, non-invasive, and a repeatable measuring approach with diagnostic and prognostic value that allows real-time monitoring.

## MATERIALS AND METHODS

### Data source and search strategy

We reviewed reports published in PubMed, EMBASE, Web of Science and the Cochrane Library. We used these searched terms: ‘*KRAS*’ or ‘GTPase KRAS’ or ‘V-Ki-ras2 Kirsten rat sarcoma viral oncogene homolog,’ ‘serum’ or ‘plasma’ or ‘circulating,’ ‘mutation,’ ‘cancer’ or ‘carcinoma’ or ‘tumor’ or ‘neoplasm,’ and ‘lung.’ Only studies published in English were included.

### Inclusion and exclusion criteria

Inclusion criteria for primary studies were d a definite diagnosis of lung cancer; *KRAS* mutations diagnosed with ctDNA, CTC, or tumor tissue; sufficient information for a 2 × 2 table. Articles were excluded if the *KRAS* mutations were not detected using tumor tissue; tumor tissues and blood samples were not matched; there was insufficient information reported to finish a 2 × 2 table; or lung cancer data were not separate from other cancer data. All selected studies were managed using EndNote X7. Studies included in our meta-analysis were assessed by two investigators independently.

### Data extraction and quality assessment

The first author's name, year of publication, country, number of patients, sex ratio, the proportion of smokers included, adenocarcinoma (AC) ratio, tumor tissue treatment, use of serum or plasma, *KRAS* mutation detection methods, and TNM stage were collected from eligible studies. Then, 2 × 2 tables were designed to show TP, TN, FP, and FN. When a *KRAS* mutation was detected by multiple methods, data for all methods were extracted, recorded, and evaluated by two investigators independently. QUADAS-2 (quality assessment of diagnostic accuracy studies 2) was used to evaluate diagnostic accuracy quality [46] using patient selection, index test, reference standard, and flow and timing.

### Statistical analysis

*KRAS* mutation status in tumor tissues was designed as a reference standard. Diagnostic numbers (TP, FP, FN, TN) were used to calculate pooled sensitivity, specificity, positive likelihood ratio (PLR), negative likelihood ratio (NLR), diagnostic odds ratio (DOR), area under the curve (AUC) and corresponding 95% confidence intervals (95% CI). PLR is calculated as: sensitivity/(1-specificity) and NLR is: (1- sensitivity)/specificity. [47, 48] DOR is a measure of the effectiveness of a diagnostic test, which is defined as PLR/NLR. [41] Summary ROC curves (SROC) and AUCs of the SROC (AUSROC) were measured.

OS was defined as the survival time from randomization and PFS was defined as the time from randomization to progression, recurrence, death or termination of follow-up. When studies did not report HRs directly, two independent investigators calculated survival data from survival curves using an Engauge Digitizer, version 4.1integrated to calculate overall HR. The threshold effect was measured by using the ROC plane, a Spearman correlation coefficient and a p-value. We evaluated race, detection methods, and tissue treatment. Publication bias was measured using a Deek's funnel plot and (*p* = 0.218) which indicated no significant bias. [49] All statistical analyses were performed using STATA software (version 12.0, STATA Corp, MIDAS module) and Meta-Disc. Quality assessment was managed with Review Manager 5.3.

## SUPPLEMENTARY MATERIALS FIGURES


