# Supplementary material for: Diagnostic and prognostic value of blood samples for KRAS mutation identification in lung cancer: a meta-analysis
Source: Oncotarget. 2017 Mar 7;8(22):36812–23. doi: 10.18632/oncotarget.15972 (PMC5482700; doi:10.18632/oncotarget.15972)
Supplement: Supplementary file 1 [file oncotarget-08-36812-s001.pdf]

Diagnostic and prognostic value of blood samples for KRAS mutation identification in lung cancer: a meta-analysis

Supplementary Material

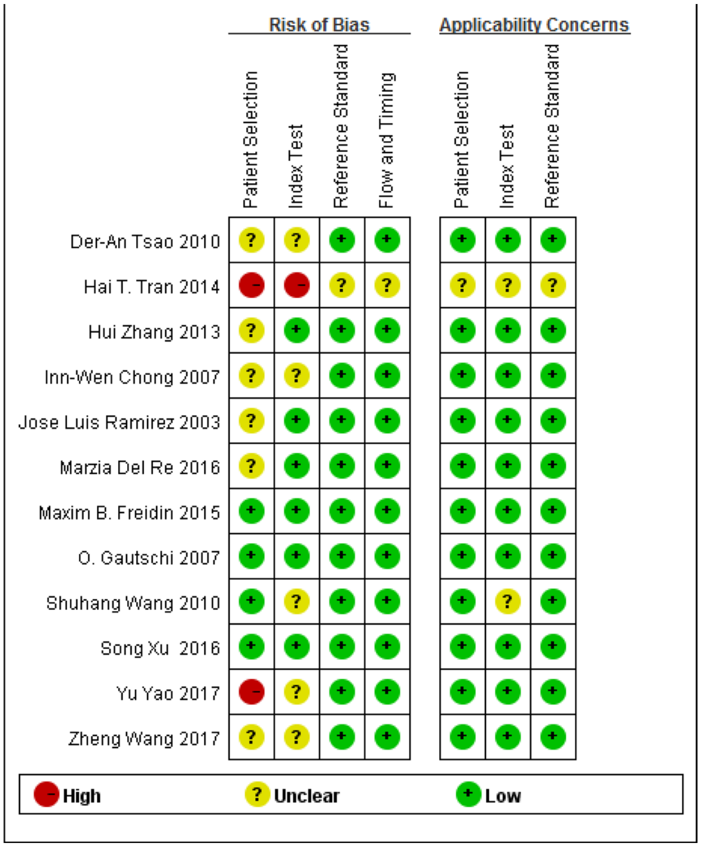

Supplementary Figure 1: QUADAS-2 plot of included studies discussing blood sample versus tumor tissue for KRAS mutation in lung cancer. Each row represents an included study. Each rank represents a different evaluation criterion. Red means high risk. Yellow means medium risk and green means low risk.

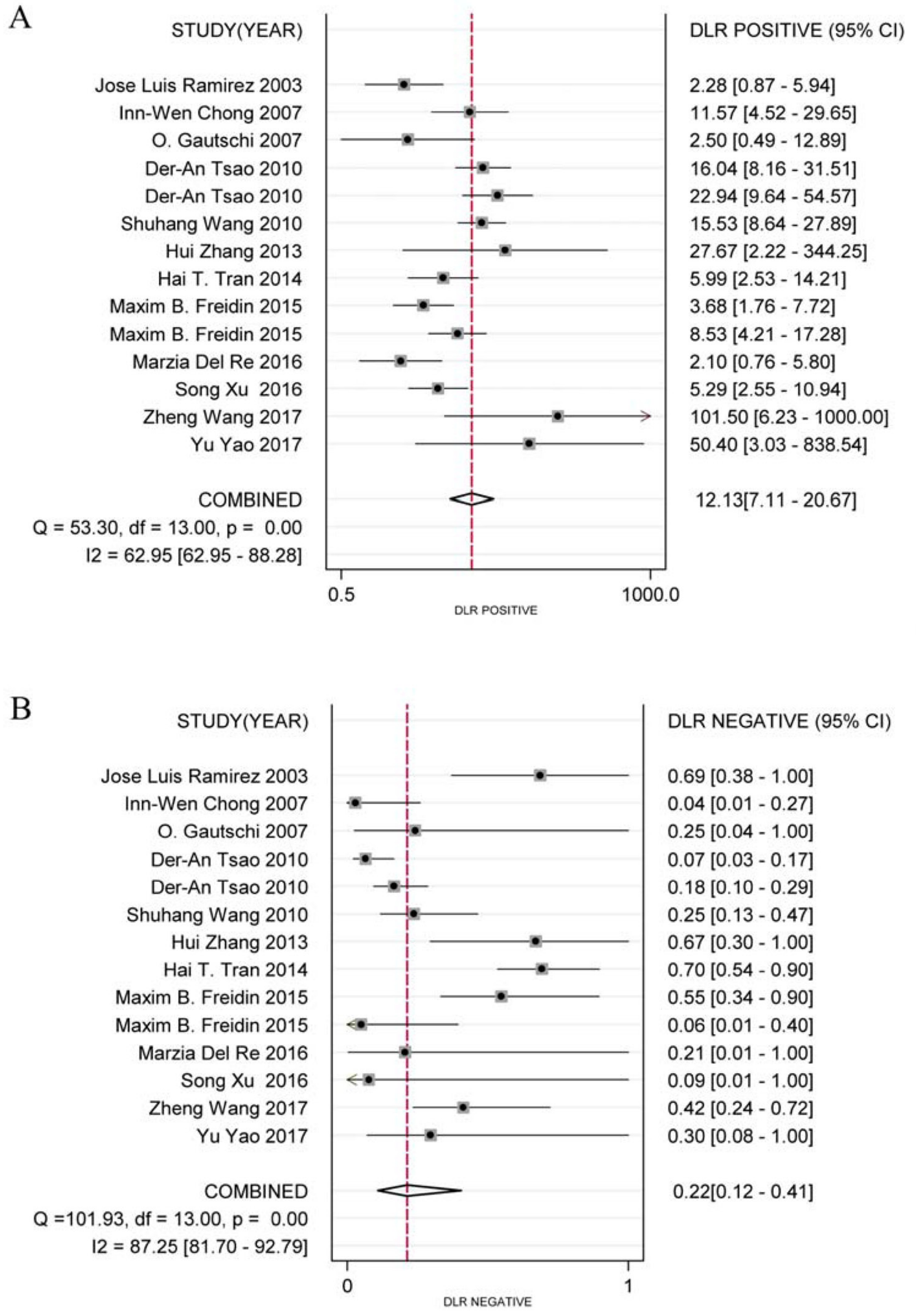

Supplementary Figure 2: Forest plots of positive likelihood ratio(A) and negative likelihood ratio(B). The width of the horizontal line represents the 95% CI of each study, square means the estimated value of every study. The diamond represents the pooled PLR, NLR and 95%CI.
